# Analysis of Toxic Heavy Metals in the Pellets of Owls: A Novel Approach for the Evaluation of Environmental Pollutants

**DOI:** 10.3390/toxics10110693

**Published:** 2022-11-16

**Authors:** Sultan Nazneen, Samidurai Jayakumar, Mohammed F. Albeshr, Shahid Mahboob, Irfan Manzoor, Jeganathan Pandiyan, Kaliyamoorthy Krishnappa, Mohan Rajeswary, Marimuthu Govindarajan

**Affiliations:** 1 Department of Zoology and Wildlife Biology, A.V.C. College (Autonomous), Bharathidasan University, Mannampandal, Mayiladuthurai 609 305, Tamil Nadu, India; 2 Department of Zoology, College of Science, King Saud University, Riyadh 11451, Saudi Arabia; 3 Department of Biology, Indiana University Bloomington, Bloomington, IN 47405, USA; 4 Post Graduate and Research Department of Zoology, ADM College for Women (Autonomous), Nagapattinam 611 001, Tamil Nadu, India; 5 Unit of Mycology and Parasitology, Department of Zoology, Annamalai University, Annamalainagar 608 002, Tamil Nadu, India; 6 Unit of Natural Products and Nanotechnology, Department of Zoology, Government College for Women (Autonomous), Kumbakonam 612 001, Tamil Nadu, India

**Keywords:** environmental risk assessment, heavy metals, toxicity, environmental contamination, conservation

## Abstract

Massive quantities of unadvisable synthetic pesticides are used in modern agricultural industries in order to increase productivity to convene food demands. Wild birds are an excellent bio-indicator of environmental contaminations as pesticides and heavy metals are intentionally highly hazardous pollutants. Considerably, raptorial birds (owls) attract consumers in the food chain and food web because they have wider forager and foraging grounds. In the current investigation, owl pellets were used as a viable tool and novel approach to detecting environmental contaminants. In total, 30 pellets comprising five species were collected from selected farmlands, and 11 metals (Cr, Mn, Co, Mo, Se, V, Cu, Ni, Pb, Zn, and Fe) were analyzed using inductively coupled plasma mass spectrometry (ICP-MS). Undeniably, the Brown Fish Owl showed more metal accumulation than the Barn Owl, Spotted Owl, Indian Eagle Owl, and Mottled Wood Owl. Among the species, the levels of metals such as Manganese (Mn), Molybdenum (Mo), Vanadium (V), Copper (Cu) and Zinc (Zn) varied significantly (*p* < 0.05). Nonetheless, the research revealed that the agroecosystem was contaminated with heavy metals. The present outcome highlights that the management of the environment, especially the agroecosystem, must be examined with a careful assessment of contaminants, and it is a vital resource for human and other related wildlife faunal communities.

## 1. Introduction

Agriculture is undoubtedly a major sector for many livelihoods. Several undesirable synthetic pollutants have been introduced due to the green revolution that empathy instigates [1]. Heavy metals are among the most harmful pollutants and cause numerous disorders in avian species [2]. However, the assessment of metals in raptorial birds, especially in the owl, has not been studied much in India. Owls are the best night hunters, and are involved in the food chain and web [3]. They are dietary generalists who mostly prefer to prey on rodents [4,5].

Consequently, they are prime and ideal pest control agents in agroecosystems. Pellets are regurgitated and spat forth by owls at their nesting sites and are made up of undigested prey items, including fur, hair, bones, skulls, etc. The pellets also include the owls’ mucus and other chemical substances found in their gastrointestinal systems. Therefore, pellet investigation is the predominant technique to determine whether the owl diets are eco-friendly and is the most ideal tool for examining environmental contaminants [6]. Considerably, owls are peak and predominant consumers in the food web, and have substantial foraging grounds. It is anticipated they will accumulate a higher concentration of toxic materials (pesticides and heavy metals) as they are present in the foraging grounds [7].

Nevertheless, raptorial birds are extremely susceptible to secondary poisoning through their prey [8]. Comprehensive study has not yet been done on metal accumulation in owls [9,10], and most of the current studies have focused on destructive samples such as the muscles, liver, brain, heart, kidneys, and bones [11]. Moreover, literature pertinent to heavy metal or toxic contaminants in raptorial birds are insufficient [11], as well as literature on metal exposure considering oxidative stress, metal toxicity [9], and metal accumulation in male and female owls [10], and metal concentrations and their impacts on breeding success [6]. Nonetheless, reports on heavy metal contamination using non-destructive samples such as pellets are inadequate [6]. Natural predators are the best remedy for pest control mechanisms and one of the best eco-friendly tools [11]. Indeed, raptorial birds (owls) are vital components in agriculture because they largely depend on rodents and insects. Certainly, importance of pest management in agroecosystems for owls has been recognised, but more relevant data are required regarding the comprehensive scientific background. Consequently, raptorial birds can be used as novel indicators and early warning tools to evaluate the harmfulness of pollutants, especially for metal contaminants. Therefore, the current invagination planned to detect heavy metal contamination in five different owl species using their pellets, as this is a non-invasive, novel, and first of its kind technique in India. This will undoubtedly facilitate conservationists and environmentalists to understand the mode of interaction and the effect of heavy metals on birds, especially raptors in farmlands. 

## 2. Materials and Methods

### 2.1. Study Area and Pellet Collection 

Opportunistic sampling was done in the agricultural landscape of the Cauvery Deltaic Zone (CDZ), which is regarded as the ‘Rice Bowl’ of South India. Agriculture is the principal occupation of the study sites. Because of the rigorous use of organic and inorganic chemicals in farming, the level of metal elements has been increasing in these areas. These accumulate in top-level predators such as owls as they feed on agricultural pests and rodents. In addition, owl species forage in agricultural aquatic habitats as feeding grounds as these agricultural habitats provide sufficient prey, such as rodents, crustaceans, reptiles, etc. In total, 30 pellets were sampled fortnightly using the roosting and breeding sites of the Barn Owl, Spotted Owlet, Indian Eagle Owl, Brown Fish Owl, and Mottled Wood Owl between December 2019 and November 2020. The sampling sites were recognized by secondary signs such as regurgitated food items, milky white stools, prey leftovers, and feathers [5,12,13]. Regurgitated pellets were wrapped in aluminium foil and were bagged in separate zip-locked covers, and the labelled covers were transported to the workroom for further investigation.

### 2.2. Assessment of Heavy Metals

The owl pellets were measured for length, breadth, and weight and dried in a hot-air oven over 24 h at 105 °C until no weight reduction occurred. Pellet remnants were ground using a homogenizer and were weighed using a weighing balance. Element residues (µg/g) in the pellets were assessed on a dry weight (DW) basis [6]. Five grams of the pellet was digested using a microwave digester (Milestone, MLS 1200) using 10 mL nitric acid (HNO_3_; 69% GR) for 10 min, 1 mL perchloric acid (HClO4; 70% GR) for 5 min. and 5 mL hydrogen peroxide (H_2_O_2_ 30% GR) for 10 min at 250 W magnetron power using the acid digestion method [14]. The digested solutions were shifted using filter paper and were kept in a deep freezer until analysis. Metals such as chromium (Cr), manganese (Mn), cobalt (Co), molybdenum (Mo), selenium (Se), vanadium (V), copper (Cu), nickel (Ni), lead (Pb), zinc (Zn), and iron (Fe) were determined using ICP-MS (inductively coupled plasma mass spectrometry). After being run in triplicate, the quantification and the average were used for further analysis. The detection limits (μg/kg d.w.) were as follows: Cr 0.1, Mn 0.1, Co 0.1, Mo 0.1, Se 0.1, V 0.1, Cu 0.1, Ni 0.1, Pb 0.1, Zn 0.1, and Fe 0.1. The recoveries were adjusted between 95 and 101 % for all of the metals analysed. 

### 2.3. Data Analysis 

The arithmetic mean and standard error were measured to compare the results of heavy metal contamination. After normality was tested (Shapiro–Wilk), one-way analysis of variance (ANOVA) was computed to validate the difference in metal concentration among species. Pearson’s correlation test was performed to understand the affiliation between length, breadth, weight, and heavy metal concentration. Statistical analysis was made with IBM-SPSS, model 25.0, and significance was *p* < 0.05, *p* < 0.001.

## 3. Results

In total, 30 pellet samples comprising five species of owls were collected and analysed for the current study. The average length, breadth, and weight of the pellets; feeding habits; and conservation status of owl species are presented in Table 1. Residues of heavy metals were noticed in 100% of the pellet samples analysed in the present study. The Brown Fish Owl showed a greater level of heavy metals such as Cr, Mg, Co, V, Ni, and Fe than the other species of owls studied. However, the Mo, Se, Cu, Pb, and Zn were the highest in the Spotted Owls compared with the other species (Table 2). Zn was the highest in the Barn Owl, Indian Eagle Owl, and Mottled Wood Owl. The Mg, Mo, V, Cu, and Zn varied significantly among the owl species compared with the other metals found in the pellets (*p* < 0.05). Indeed, the level of various metals examined in the five different species of owl was as follows: Fe > Mg > V > Cr > Ni > Co > Zn > Cu > Pb > Se > Mo. 

Iron (Fe) was found to be in the highest proportion, making up more than 90 % of all heavy metals, with molybdenum (Mo) contributing the least. In addition, a positive association was found between the length, width, and weight of the pellets and the various metal components when a Pearson correlation test was performed (Table 3). Similarly, the Cr level showed positive correlation with Mg (r = 0.836; *p* < 0.05) (Table 3). Equally, Co had a positive association with Cr and Mg. Likewise, the Se concentration in the pellets showed positive interaction with Mo. In addition, the V concentration detected in the pellets showed a strong positive correlation with Cr (r = 0.941; *p* < 0.05), Mg (r = 0.899; *p* < 0.05), and Co (r = 0.926; *p* < 0.05). Similarly, Cu displayed a strong positive correlation with Cr, Mg, Co, Mo, Se, and V. The concentration of Ni in the pellets showed a positive interaction with the concentration of Cr, Mg, Co, V, and Cu. Pb showed a positive association with Mo and Cu. Zn was the only element positively associated with all heavy metals such as Cr, Mg, Co, Mo, Se, V, Cu, Ni, Pb, Zn, and Fe. Similarly, Fe displayed a positive interaction with Cr, Mg, Co, V, and Ni (Table 3).

## 4. Discussion

Free-ranging avian species are one of the most significant indicators for assessing the quality of environmental pollution and are non-targeted victims as they are frequently exposed to different contaminants in various directions [7,8]. In India, very little research has been done regarding pollutants and their effects on wild birds [2,15,16]. The results of the current study found that the heavy metals in the five species of owl pellets showed relatively higher concentrations than the Eurasian Eagle Owl pellets, especially for lead (Pb) (0.02 to 0.04 μg/kg), chromium (Cr) (0.01 to 0.02 μg/kg), copper (Cu) (0.03 to 4.65 μg/kg), manganese (Mn) (0.06 to 16.2 μg/kg), zinc (Zn) (0.68 to 75.8), and iron (Fe) (14.1 to 234.0 μg/kg) [6]. The source and accumulation of metals might have been from the owls’ diets. Several studies have reported on the concentration of metals in owls using their blood samples, such as Eurasian Eagle Owl, Griffon Vulture, Common Buzzard, Little Owl, and Barn Owl, as well as from tissues samples for Eurasian Eagle Owls, Brown Hawk Owls, and Collared Scops Owls, and these studies found that this accumulation of metals was from their diets [9,10,17,18,19,20,21].

Studies have stated that higher concentrations of Cr have plenty of side effects in avian species, such as poor health ability, lesser productivity, uneven growth, abnormal embryo development, lesser viability, and stunted birds’ growth [22,23,24,25]. Nevertheless, a more significant accumulation of metals could affect avian species through several illnesses in their physiology, as well as their behaviour [17]. The present investigation showed that the Cr concentration in the pellets of the Brown Fish Owl (14.5 μg/kg), Barn Owl (7.4 μg/kg), India Eagle Owl (5.0 μg/kg), and Spotted Owl (6.2 μg/kg) was higher than the levels known to cause abnormalities, and thus it could be viewed as very harmful to the owl species. The metal concentration of Mn in the pellets of the owl species studied here coincided well with earlier research works. Mn is a vital element for growth and development, but indiscriminate use and continued exposure could pose negative effects on avian species [5]. The acute toxic level of Mn (21.3–469.5 mg/kg) creates muscular and hypodermal tissues, interrupting avifauna’s immune cells [26,27]. 

The level of cobalt (Co) detected from the pellets of the owl species here were lower when compared with earlier reports. The toxic effect of Co in avian fauna is understood as being 50–500 parts/106 [28], and a slow progress rate has also been noted among weaklings [3,29]. The selenium (Se) concentration in the pellets of the owl species was not much more compared with previous reports; however, Se could also exhibit a negative effect (29 mg/kg of Se in the liver), reproductive failure (15 mg/kg in the liver), malfunctioning of the immune system (5 mg/kg of Se in the liver), and hatching and distorted embryos (above 3 ppm) [30,31]. Vanadium (V) with 10 μg/g d.w. in the liver and 25 μg/g d.w. in the kidney was linked with a high mortality in avian communities [32]. Cu is indispensable for normal body functions, and is perhaps deadlier to animals and organ systems at elevated concentrations [10,33]. High levels of Cu and its possible effects have been well-defined in wild birds, without showing any symbol of fatality [10]. Furthermore, the acute effect of Cu in Canadian Geese (187–323 mg/kg) in their liver tissue is usually controlled by homeostasis with 50 µg/g, and loss of liver regulation and metabolism (95.02 µg/g), [34,35]. In addition, the Cu levels recorded in this study were found to be lower than this, and were thus not associated with those effects among birds. 

A higher concentration of nickel (Ni) in avifauna provides different biological effects: in their diet it shows various biological effects in the chicks of avifauna (300 to 800 mg/kg), as well as death and reproductive failures (1200 mg/kg) [36]. The Ni levels found in this observation were significantly lower, and were consequently not linked with those effects among avifauna. Similarly, birds are the best models for Pb toxicity [37,38] and a study proved the Pb-produced calcification in marrow cells in Canadian Geese [39]. The increasing lead concentration afforded diverse biological affects in avifauna: diarrhea, anorexia, and depression (above 6 ppm), as well as toxic/lethal effects (liver 2.0 µg/g and kidney > 2.0 µg/g) [40,41,42]. The Pb levels in the Spotted Owl and Barn Owl pellets in the present study are likely to have lethal effects. Henceforth, ornithologists are concerned about finding possible answers regarding Pb usage and toxicity. 

Janaydeh et al. [43] reported the Zn level in the muscle (44.24 ppm), liver (156.44 ppm), and kidney (61.94 ppm) of House Crow sampled from Malaysia. Similarly, Horai et al. [44] considered the level of Zn in the muscle (58.9 ppm), liver (79.1 ppm), and kidney (142 ppm) of Jungle Crow collected from Japan. Furthermore, they reported on the levels of Zn in the muscle (54 ppm), liver (122 ppm), and kidney (75.3 ppm) of Carrion Crow. Rajamani and Muralidharan [45] reported the levels of Zn in White-Rumped Vultures in India, ranging from 4.61 to 30.96 mg/kg in the kidney, 35.91 to 42.52 mg/kg in the liver, and 21 to 21.95 mg/kg in muscle. Unexpectedly, the Zn concentration noted in five species of owl in the present study showed a higher concentration than previously. In birds, the following levels exist, namely the threshold level of zinc (1200 µg/g), poisoning signs of Zn concentrations (840.0 and 1410.0 μg/g), and Zn toxicity (525.0 μg/g) in the liver tissue [46,47,48,49]. Fe is the most vital element abundant in some food items and is essential for the well-being of animals, including birds. Most of the pellet samples showed the highest concentration of Fe, which would likely cause ill effects on birds [38]. The study found an inter-correlation among the metals and the results indicated that the metals have inter-relationships that influence their concentration in avian species [50,51,52]. 

**Figure 1:**
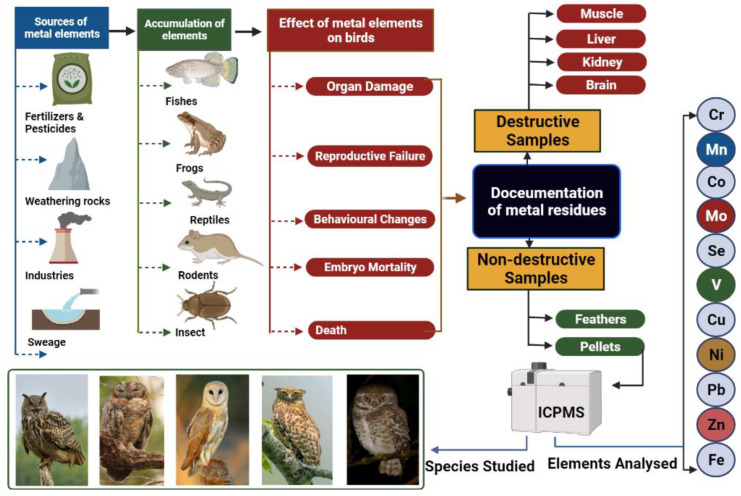
Impacts of metals on birds and the mechanisms behind them.

In fact, the sources, accumulation of metals, and their effects on various parts of the avian species are clearly discussed in Figure 1. 

Nonetheless, the predators’ metal build-up depends on the quality of the food supplies available to them in a particular ecosystem. Kim and Ho [10] reported that the accumulation of the metal concentration is proportionally interlinked with the quality of the habitat. In diet selection, owl species with a higher concentration of heavy metals prey on frogs, freshwater crabs, snakes, lizards, and rodents mainly inhabiting agricultural ecosystems, where unadvisable fertilizers and pesticides have been used. Non-required elements in food have a unique value in various tissues of birds, including regurgitated pellets, and could be predicted to have different metal build-up among species with diverse food sources [51,53,54]. 

Barn Owl pellets have been shown to be positively linked with metals based on their features (Table 3). The Barn Owl pellet weight was significantly correlated with both pellet length and width, but pellet width was positively correlated with pellet length (*p* < 0.001). However, the concentration of metals and other chemicals may depend on the pellets’ size and weight. Except for Cr, all of the elements found in Barn Owl pellets exhibit positive correlations. Several sources, including bird feathers, waterbird organs, and other sources, have been studied for their metal–chemical interdependence [10,14,16]. Metals are interdependent, as shown by the findings obtained in the present research; hence, a positive association was identified among the metals analysed from the Barn Owl pellets.

Most of the owls in the study are known to forage, roost, and breed in hillocks and other suitable microhabitats such as Palmyra trees and small caves in the vicinity of agroecosystems. Increased use of fertilizers and pesticides, urban development, alternation of human settlements, and other anthropogenic pressures could be the main sources of metal pollution in these sites. Nevertheless, the present study’s findings could be helpful for the government sectors, especially agriculture, civic health, pollution control, and wildlife management and conservation, to set up informed guidelines and make policy decisions on the rational use of pesticides and fertilizers. Such measures could help protect wild birds, including owls and their prey species. 

## 5. Conclusions

Heavy metal contamination was found in pellets from all five Barn Owl species. Nonetheless, the results demonstrate that Barn Owls’ prey species have harmful elements, as measured by heavy metal levels that are elevated over a threshold. Barn Owls, which often forage in agricultural areas, leave behind pellets that may provide important information about the kind of food they eat and where they found it, which might lead to improved raptor management and conservation in the future.

## Figures and Tables

**Table 1:** Various owl pellets collected from the farms of the Cauvery Deltaic regions.

Common Name	Scientific Name	Morphometric Measurements of Pellet (Mean ± SE)			Feeding Habit ^a^	Conservation Status ^b^
		Length (cm)	Breadth (cm)	Weight (g)		
Barn Owl	*Tyto alba*	4.6 ± 0.8	2.9 ± 0.2	3.7 ± 0.5	Chiefly small mammals also take bats, small birds, lizards, amphibians, and insects.	Least Concern, Population stable
Brown Fish Owl	*Bubo zeylonensis*	5.1 ± 0.5	3.4 ± 0.4	7.3 ± 1.6	Mainly fish, frogs and freshwater crabs and eat crayfish, snakes, lizards, and occasionally rodents, and birds.	Least Concern, Population decreasing
Indian Eagle Owl	*Bubo bengalensis*	5.9 ± 0.9	3.1 ± 0.2	7.8 ± 1.5	Chiefly small mammals, also take small birds, and lizards.	Least Concern, Population stable
Mottled Wood Owl	*Strix ocellata*	3.8 ± 0.5	2.2 ± 0.2	2.8 ± 0.6	They feed on palm squirrels, mice, and other small mammals.	Least Concern, Population unknown
Spotted Owl	*Athene brama*	4.3 ± 0.5	1.7 ± 0.2	1.3 ± 0.3	It hunts a variety of insects and small vertebrates.	Least Concern, Population stable

^a, b^ Source: Birdlife International species factsheet (http://www.birdlife.org). Assessed on 22 October 2021.

**Table 2:** Concentration of heavy metals (μg/kg, mean± SE) recorded in five different species of owl.

Heavy Metals	Barn Owl (*n* = 6)	Brown Fish Owl (*n* = 6)	Indian Eagle Owl (*n* = 6)	Mottled Wood Owl (*n* = 6)	Spotted Owl (*n* = 6)	One way ANOVA	
						F Value	*p*-Value
Chromium	7.4 ± 2.3	14.5 ± 6.1	5.0± 2.3	3.6 ± 1.8	6.2 ± 2.9	1.534	>0.05
Manganese	26.6 ± 5.9	62.0 ± 18.0	10.8 ± 2.8	23.9 ± 8.7	35.7 ± 12.4	3.045	<0.05
Cobalt	0.6 ± 0.3	1.7 ± 0.7	0.2 ± 0.1	0.5 ± 0.4	0.7 ± 0.5	1.623	>0.05
Molybdenum	0.1 ± 0.0	BDL	BDL	0.1 ± 0.1	0.8 ± 0.4	3.212	<0.05
Selenium	0.4 ± 0.2	0.2 ± 0.1	0.1 ± 0.1	0.6 ± 0.3	1.5 ± 0.9	1.637	>0.05
Vanadium	4.7 ± 1.5	15.0 ± 5.0	2.6 ± 1.2	2.8 ± 1.4	4.4 ± 3.1	3.321	<0.05
Copper	6.6 ± 2.2	9.6 ± 4.5	0.9 ± 0.6	1.8 ± 1.1	19.3 ± 7.8	3.112	<0.05
Nickel	3.8 ± 1.0	11.7 ± 5.6	2.3 ± 0.9	3.4 ± 1.7	2.6 ± 1.7	2.010	>0.05
Lead	2.2 ± 1.1	0.3 ± 0.2	BDL	1.3 ± 0.6	6.9 ± 3.9	2.294	>0.05
Zinc	237.3 ± 40.3	176.7 ± 51.7	98.9 ± 43.8	182.3 ± 17.3	358.7 ± 83.0	3.456	<0.05
Iron	3075.6 ± 834.5	10,498.2 ± 5919.7	1193.4 ± 494.5	1699.4 ± 627.0	2912.4 ± 1370.3	1.874	>0.05

**Table 3:** Pearson correlation showing the relationships among the metal collected from the five different species of owls from the farmlands of the Cauvery Deltaic region.

	Pellet Length	Pellet Breadth	Pellet Weight	Cr	Mn	Co	Mo	Se	V	Cu	Ni	Pb	Zn
Pellet breadth	0.482 **												
Pellet weight	0.687 **	0.597 **											
Cr	−0.243	0.119	−0.047										
Mn	−0.310	−0.074	−0.108	0.836 **									
Co	−0.279	−0.034	−0.089	0.916 **	0.920 **								
Mo	−0.111	−0.387 *	−0.290	0.112	0.127	0.131							
Se	−0.119	−0.417 *	−0.289	0.258	0.335	0.335	0.422 *						
V	−0.208	0.184	0.010	0.941 **	0.899 **	0.926 **	0.070	0.279					
Cu	−0.160	−0.280	−0.327	0.573 **	0.549 **	0.560 **	0.795 **	0.636 **	0.558 **				
Ni	−0.252	0.128	−0.032	0.941 **	0.867 **	0.898 **	−0.010	0.157	0.941 **	0.469 **			
Pb	−0.111	−0.343	−0.311	0.107	0.129	0.104	0.914 **	0.332	0.069	0.754 **	0.017		
Zn	−0.280	−0.471 **	−0.468 **	0.436 *	0.532 **	00.449 *	0.509 **	0.616 **	0.409 *	0.733 **	0.377 *	0.563 **	
Fe	−0.073	−0.014	0.067	0.444 *	0.502 **	0.563^**^	0.032	0.123	0.525 **	0.265	0.427 *	0.036	0.116

** Correlation is significant at the 0.01 level (2-tailed); * correlation is significant at the 0.05 level (2-tailed).

## Data Availability

Not applicable.
